# Three-Dimensional-Printed Molds from Water-Soluble Sulfate Ceramics for Biocomposite Formation through Low-Pressure Injection Molding

**DOI:** 10.3390/ma16083077

**Published:** 2023-04-13

**Authors:** Daniil Golubchikov, Pavel Evdokimov, Dmitry Zuev, Yaroslav Filippov, Tatiana Shatalova, Valery Putlayev

**Affiliations:** 1 Department of Materials Science, Lomonosov Moscow State University, Building, 73, Leninskie Gory, 1, 119991 Moscow, Russia; zuev.dmitri@gmail.com (D.Z.); shatalovatb@my.msu.ru (T.S.); valery.putlayev@gmail.com (V.P.); 2 Department of Chemistry, Lomonosov Moscow State University, Building, 3, Leninskie Gory, 1, 119991 Moscow, Russia; pavel.evdokimov@gmail.com (P.E.); filippovyy@my.msu.ru (Y.F.); 3 Kurnakov Institute of General and Inorganic Chemistry, Russian Academy of Sciences, Leninskii Prosp., 31, 119071 Moscow, Russia; 4 Research Institute of Mechanics, Lomonosov Moscow State University, Michurinsky, 1, 119192 Moscow, Russia

**Keywords:** sulfate ceramics, low pressure injection molding, water-soluble molds, 3D printing, regenerative medicine, osteoconductivity, macroporous ceramics

## Abstract

Powder mixtures of MgSO_4_ with 5–20 mol.% Na_2_SO_4_ or K_2_SO_4_ were used as precursors for making water-soluble ceramic molds to create thermoplastic polymer/calcium phosphate composites by low pressure injection molding. To increase the strength of the ceramic molds, 5 wt.% of tetragonal ZrO_2_ (Y_2_O_3_-stabilized) was added to the precursor powders. A uniform distribution of ZrO_2_ particles was obtained. The average grain size for Na-containing ceramics ranged from 3.5 ± 0.8 µm for MgSO_4_/Na_2_SO_4_ = 91/9% to 4.8 ± 1.1 µm for MgSO_4_/Na_2_SO_4_ = 83/17%. For K-containing ceramics, the values were 3.5 ± 0.8 µm for all of the samples. The addition of ZrO_2_ made a significant contribution to the strength of ceramics: for the MgSO_4_/Na_2_SO_4_ = 83/17% sample, the compressive strength increased by 49% (up to 6.7 ± 1.3 MPa), and for the stronger MgSO_4_/K_2_SO_4_ = 83/17% by 39% (up to 8.4 ± 0.6 MPa). The average dissolution time of the ceramic molds in water did not exceed 25 min.

## 1. Introduction

The application of regenerative medicine approaches for the restoration of damaged bone tissue [1,2,3,4,5] requires appropriate materials for creating scaffolds, among which ceramic [6,7,8,9,10,11] and composite materials [12,13,14,15] are widely used. The requirements for materials to manufacture scaffolds (implants) for bone regeneration include (i) biocompatibility [16,17,18], (ii) bioresorbability (the material must completely or partially degrade/dissolve, freeing up space for bone tissue de novo) [19,20], (iii) mechanical compatibility with bone [21,22], (iv) good wettability, (v) osteoinductivity (facilitating the growth of bone tissue not only at the interface of the native bone/implant contact, but also in the entire volume of the porous scaffold) [23], and (vi) osteoconductivity (growing of the bone into the porous scaffold with the ingrowth of a network of blood vessels, as well as maintaining the flow of extracellular fluid through the implant) provided by the porous architecture of the implant/scaffold [2,24,25,26,27,28,29].

Composites with a matrix based on natural and synthetic polymers are promising materials for scaffold formation [30]. Among the latter, polyesters such as polylactide (PLA) [31], polycaprolactone (PCL) [32], and polyhydroxybutyrate (PHB) [33,34] filled with various phosphates, e.g., hydroxyapatite (HA) or tricalcium phosphate (TCP), are especially distinguished. The listed polymers are biodegradable and have a sufficient mechanical strength, which leads to the ability to withstand the necessary mechanical loads. Nevertheless, the surface of such materials is essentially hydrophobic. The addition of the phosphate particles leads to further improvement in the mechanical characteristics of the material [35]. Furthermore, the particles on the surface make the scaffolds more hydrophilic. To ensure osteoconductive properties, scaffolds must have a system of interconnected pores with a diameter of about 100–500 µm [36], which form a special macrostructure (architecture) of a macroporous scaffold.

There are two fundamental approaches for the creation of macroporous composite implants (scaffolds) with a given architecture. The fused deposition modelling (FDM) technique allows for performing direct 3D printing from thermoplastic polymers [37], which are pure or filled with an inorganic phosphate filler. However, the lateral resolution of this method does not exceed 100 µm [38]. An alternative approach is a replica technique [39]. In fact, such a technique is identical to casting into a sacrificial mold with a special architecture. The latter can be produced using additive technologies with a suitable resolution [38,40], e.g., using stereolithographic 3D printing. The sufficient resolution can be achieved either through direct printing with a photocurable monomer filled with ceramic precursor particles, followed by debinding of the polymer matrix. Otherwise, the preform can be printed from the unfilled photopolymer, followed by filling the preform with a ceramic slurry. Furthermore, the polymer preform is burned out (or dissolved) to form a macroporous ceramic structure, which is a sacrificial ceramic mold for the subsequent casting of a filled thermoplastic polymer composite into it [41].

The choice of the material to create a soluble form is challenging. In addition to the strength characteristics that are necessary for low pressure injection molding (LPIM), the mold material should not greatly increase in volume when dissolved. Otherwise, volumetric effect can cause destruction of the target thermoplastic mold. In this work, ceramics made of magnesium sulfate was chosen to manufacture the sacrificial mold. Despite the fact that sulfate ceramics have been described very little in existing studies, it could be a promising material for creating the above-described soluble forms [42]. There are several studies on the creation and application of sulfate and oxosulfate 3D structures as supporting materials [43]. The presented results indicate sufficient mechanical strength [44] required for the injection molding of a polymer into such a mold. The possibilities of using calcium carbonate [45], magnesium sulfate [44], and graphite [46] as a material for creating a supporting structure for the formation of suspended structures using 3D printing methods have also been described. The removal of such materials can be carried out by oxidative roasting (in the case of graphite), as well as by dissolution in water or acid. Magnesium-sulfate-based ceramics have been confirmed to have an acceptable bending strength and can dissolve rapidly in water [44].

The aim of this work was to develop and create soluble sacrificial molds from sulfate ceramics for the manufacture of macroporous scaffolds based on filled thermoplastics using the LPIM technique. In the current work, a technique for creating sulfate ceramics with the composition MgSO_4_/(Na or K)_2_SO_4_ to manufacture soluble “sacrificial” forms has been developed. The mechanical and structural properties of the obtained compositions have also been studied. To improve the sintering and strength properties of sulfate ceramics, an admixture of nanodispersed tetragonal zirconia was added to the charge composition.

## 2. Materials and Methods

### 2.1. Materials

Powders of magnesium sulfate (Sigma Aldrich, Product of Japan, CAS: 7487-88-9, Tokyo, Japan), sodium sulfate (Sigma Aldrich, Product of India, CAS: 7757-82-6, Mumbai, India), potassium sulfate (Ruskhim, Moscow, Russia), and zirconium dioxide (3% yttrium oxide-doped (Y-TZP), Y_2_O_3,_ donated by Baikov Institute of Metallurgy and Materials Sciences, Russian Academy of Sciences, Moscow, Russia) were used to prepare the powder mixes.

### 2.2. Powder Mixtures Preparation

The target phase compositions of the sulfate ceramics are presented in Table 1. Sintering temperatures for K-containing ceramics (T = 850 °C) [47] and Na-containing ceramics (T = 800 °C) [48] were selected based on the corresponding phase diagrams.

In total, 20 g of starting components, which are presented in Table 1, as well as 100 g of grinding balls (zirconium dioxide, d = 6.8 mm) and 10 mL of acetone, were placed into containers made of stabilized zirconium oxide. Containers with the described components were fixed in the planetary mill (Fritch Pulverisette, Idar-Oberstein, Germany). The mixing of magnesium sulfate MgSO_4_ and sodium (potassium) sulfate Na(K)_2_SO_4_ with 5 wt.% of Y-stabilized tetragonal zirconium dioxide ZrO_2_ (Y-TZP samples line) was conducted for 10 min at a rotation speed of 120 rpm. The soft milling mode was chosen to prevent the appearance of monoclinic zirconium dioxide (debris from containers during milling). After milling, the powders were dried and passed through a sieve with 200 μm mesh.

### 2.3. Ceramic Samples Preparation

Powder compacts in the form of cylinders with a diameter of 6 mm and height of 12 mm were pressed at 20 MPa for 1 min in a steel die using the manual press (Carver Laboratory Press model C, Fred S. Carver, Inc., Wabash, IN, USA). The compacts were sintered in the air at 800 °C (Na-containing) and 850 °C (K-containing) and held with a dwell-time of 6 h with a heating rate of 5°/min to reach the temperature of sintering.

### 2.4. 3D Molds Preparation

**Figure 1:**
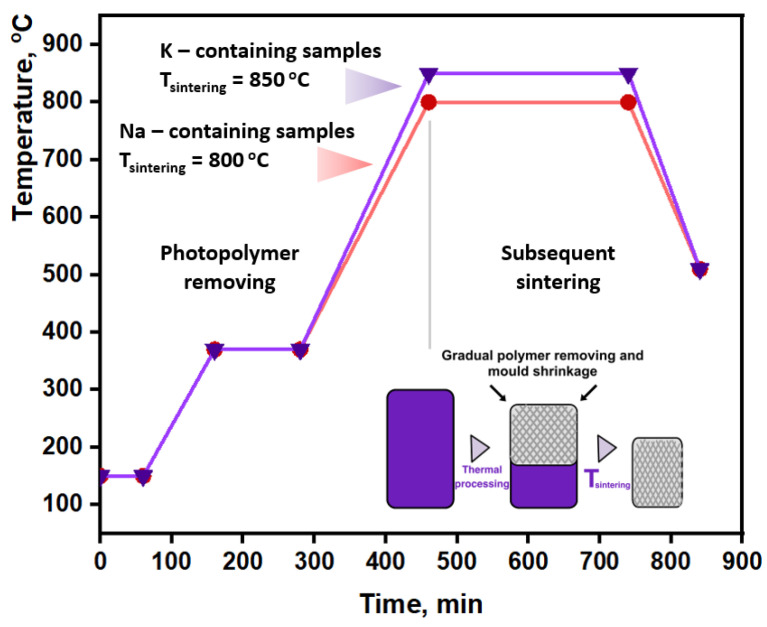
Time−temperature schedule to burn out photocured resin and to sinter ceramic residue.

The sulfate powder mixture was dried for 24 h at a temperature of 60 °C and was mixed with 1 vol.% of Triton X-100 (2-[4-(2,4,4-trimethylpentan-2-yl)phenoxy] ethanol, Sigma Aldrich, CAS: 93443, Saint Louis, MO, USA) in a planetary mill for 1 h at a rotation speed of 120 rpm. To obtain a photocurable suspension, 30 vol.% of the sulfate powders were mixed with Triton X-100 and they were mixed with a light-hardening commercially available polymer (mixture of acrylic monomers and oligomers) by ball milling through seven cycles of 1 min each at a speed of 3200 rpm. The mass ratio was 30/70. Kelvin-shaped structures (height of 5 cm, diameter of 1 mm, and porosity of 60%) made from Castable Wax Resin (Formlabs, Boston, MA, USA) by stereolithography 3D printing (Amber, Autodesk, San Francisco, CA, USA) were impregnated with the above-mentioned suspensions under vacuum. To implement the burnout process, the Standard Burnout Schedule, commercial scheme, was adapted [49] and used (Figure 1).

### 2.5. Characterization Techniques

The bulk density of the samples after heat treatment was calculated using Equation (1). The calculated density was defined according to Equation (2).

*ρ_bulk_* = *m*/(*h* × π*D*^2^/4), (1)

*ρ_calculated_* = *n*_1_ × *ρ*_1_ + *n*_2_ × *ρ*_2_, (2)

where:

*ρ*—density of the sample, g/cm^3^;
*m*—weight of the sample, g;
*h*—thickness of the sample, cm
*D*—diameter of the sample, cm;
*ρ_n_*—density of the corresponding phase, g/cm^3^;
*n_n_*—the content of the corresponding phase.

The mass and the linear dimensions of the samples were measured with an accuracy of ±0.001 g and ±0.01 mm, respectively, before and after the heat treatment.

Thermal analysis (TA) including thermogravimetry (TG) and differential thermal analysis (DTA) was performed using an STA 409 PC Luxx thermal analyzer (NETZSCH, Selb, Germany) during heating in air (10 °C/min, 40–1000 °C), the specimen mass was at least 10 mg. The gas-phase composition was monitored with a Netzsch QMS 403C Aëolos quadrupole mass spectrometer (NETZSCH, Selb, Germany) coupled with a Netzsch STA 409 PC Luxx thermal analyzer (NETZSCH, Selb, Germany). The mass spectra were recorded for the following *m*/*z* values: 18 (H_2_O); 30 (NO).

The phase composition of the powders obtained after the synthesis was determined by X-ray powder diffraction (XRD) using a Rigaku D/Max-2500 diffractometer (Rigaku Corporation, Tokyo, Japan) with a rotating anode (CuKα radiation). Phase identification was performed using the ICDD PDF2 database and literature data.

Dilatometric analysis was carried out using a DIL 402 C horizontal dilatometer (Netzsch, Germany). The specimens were heated at a rate of 5 °C/min to temperatures between 750 and 800 °C.

The compressive strength of the prepared ceramics was measured using cylindrical samples with a diameter/height ratio of 1:2 (diameter of 8 mm and height of 16 mm). The samples were subjected to uniaxial compression with a universal test machine P-05, equipped with multi-channel measuring system (Spider, Germany), along the cylinder axis at a crosshead rate of 1 mm/min.

Scanning electron microscopy (SEM) images, elemental maps, and linescans of the obtained ceramics were done with SEM on a LEO SUPRA 50VP FEG electron microscope (Carl Zeiss, Jena, Germany) at an accelerating voltage of 3–20 kV using an SE2-detector. A chromium layer (≤10 nm in thickness) was sputtered onto the surface of the ceramic sample (Quorum Technologies spraying plant, Q150T ES, Great Britain, London, UK). Back-scattered QBSD, SE2-type, BSE detectors were used.

The measurement of the grain sizes was carried from SEM images by measuring the area of the grain in ImageJ free software (Version 1.53t, U.S. National Institutes of Health, Bethesda, MD, USA) and the radius of the circle inscribed to the corresponding area. To construct the histograms of the grain distributions, at least 100 particles were measured each time.

## 3. Results and Discussion

There are several problems related to the sulfate ceramic sintering. Firstly, the sulfates can be easily reduced by carbon residues during sintering, which leads to sulfide formation. This is the origin of low-fusible eutectics and cracking of the samples. Another issue is the low diffusion rate of the tetrahedral sulfate anion, which impedes effective mass transporting. This situation also occurs in similar compounds with tetrahedral anions, such as phosphates [50,51] and silicates [52].

The above issues lead to difficulty in the solid-state sintering of sulfate ceramics and force the application of sintering temperatures close to the melting point of the corresponding sulfates. The alternative option is liquid phase sintering (LPS) [53].

**Figure 2:**
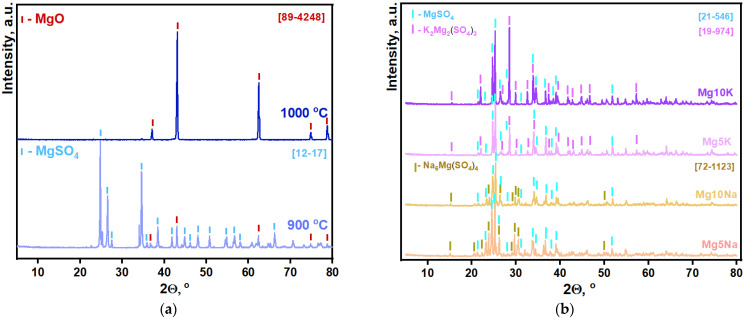
XRD data of the samples: (**a**) MgSO_4_ after firing at 900 and 1000 °C; (**b**) Mg5K and Mg10K after firing at 850 °C, and Mg5Na and Mg10K after firing at 800 °C.

The sintering of pure magnesium sulfate at 900 °C led to crumbly and fragile samples (according to XRD, Figure 2a and Figure S1, the samples were still single-phase), whereas sintering at 1000 °C led to the conversion of magnesium sulfate to magnesium oxide. Thereby, dense and tough sulfate ceramics cannot be produced by the solid-state sintering of MgSO_4_. To conduct LPS, eutectic forming additives of sodium sulfate or potassium sulfate to MgSO_4_ can be used. Based on the corresponding MgSO_4_-K_2_SO_4_ (T_Mg-rich eutectics_ = 899 °C, 77.9 mol.% of Mg) [47] and MgSO_4_-Na_2_SO_4_ (T_Mg-rich peritectics_ = 807 °C, 72.2 mol.% of Mg) [48] phase diagrams, we decided to prepare samples with 5% (Mg5K, Mg5Na), 10% (Mg10K, Mg10Na), 15% (Mg15K, Mg15Na), and 20% (Mg20K, Mg20Na) of M_2_SO_4_ (M = K, Na). According to the XRD data after sintering at 850 °C (Figure 2b), MgSO_4_ and K_2_Mg_2_(SO_4_)_3_ (Langbeinite) phases were presented in the samples from the MgSO_4_-K_2_SO_4_ system. MgSO_4_ and Na_6_Mg(SO_4_)_4_ phases were obtained in the samples from the MgSO_4_-Na_2_SO_4_ system after sintering at 800 °C. A higher content of mixed Mg_x_M_y_SO_4_ phases was obviously obtained in the Mg10M sample in comparison with the Mg5M sample (M = K, Na). All sintered ceramic samples had a high density and toughness that was necessary to withstand mechanical stress during the realization of the low pressure injection molding (LPIM) process.

**Figure 3:**
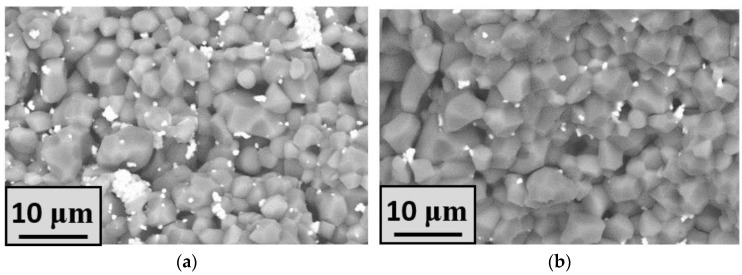

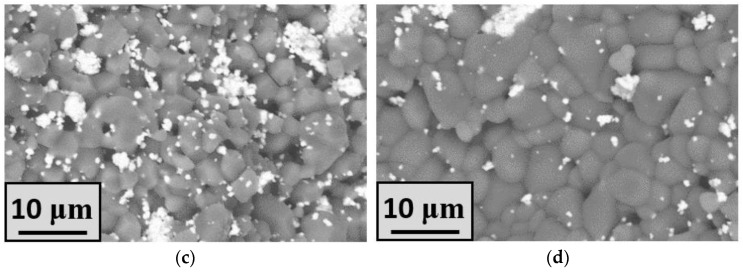
SEM images (QBSD) of the samples: (**a**) Mg5KY-TZP, (**b**) Mg15KY-TZP, (**c**) Mg5NaY-TZP, and (**d**) Mg15NaY-TZP.

The all-sintered ceramic samples had a high density and toughness, which is necessary to withstand mechanical stress during low-pressure injection molding (LPIM). The strength of the obtained sulfate ceramics was noticeably improved by the addition of zirconia powder. The effect of the addition of zirconia particles was occasionally observed for the samples made from sulfate powders and milled in zirconia milling containers for 1 h at a rotation speed higher than 600 rpm. After this effect was first discovered, the tetragonal zirconia phase was added intentionally to the sulfate ceramic precursors to initiate the deceleration of crack propagation and the transformational toughening of the samples [54]. Moreover, the grain growth suppression due to the effect of grain boundary pinning by small inclusions during sintering could be achieved [55]. The addition of tetragonal zirconia significantly improved the mechanical characteristics of various composites [56,57]. The detection of the zirconia particles in the bulk of the ceramics was possible with SEM using a back-scattered (BSE) detector. Lighter areas corresponded to a higher content of heavier chemical elements such as zirconium. Therefore, darker areas of grains in the SEM images (Figure 3) corresponded to K_2_Mg(SO_4_)_3_ or Na_6_Mg(SO_4_)_4_ phases. Ceramics sintered with the addition of zirconium dioxide were dense and contained an insignificant number of pores. There was no significant difference between grain size in the Mg5K and Mg15K samples; however, the density was slightly increased with the increase in potassium content. The Na-containing samples also tended to be densified with the increase in the amount of sodium. It is worth noting the grain growth, especially in the areas with a lack of the zirconia particles.

**Figure 4:**
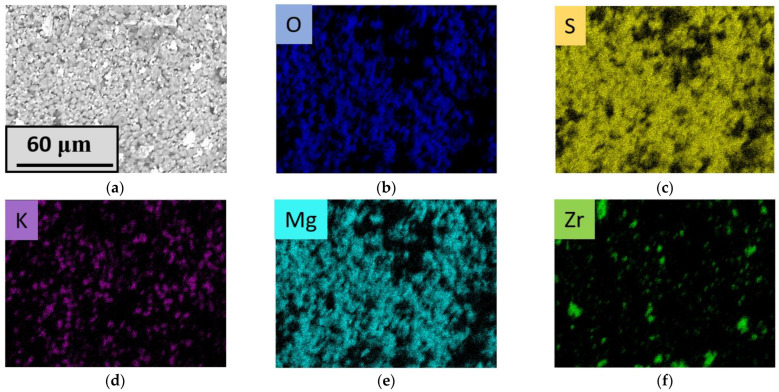
(**a**) SEM image of Mg10KY-TZP; (**b**–**f**) corresponding elemental maps.

To provide more clear imaging of the elemental distribution, EDX mapping was accomplished. Elemental mapping data for the Mg10K sample is shown at Figure 4. Sulfate phase homogeneity was discovered from the sulfur and oxygen elemental maps, as these elements occurred in all of the phases obtained (excluding the absence of the sulfur in zirconium oxide). It was observed that potassium-containing grains were presented in a sufficient part of the studied area, except for pore areas with diameters of 2–5 μm. It is worth noting that potassium-containing grains were matched with the areas where magnesium was found. This means that the K_2_Mg(SO_4_)_3_ phase formed individual grains with an average diameter of 3–5 μm. Zirconium dioxide particles are colored green at Figure 4f and can be matched with the black zones in the sulfur map (Figure 4c).

**Figure 5:**
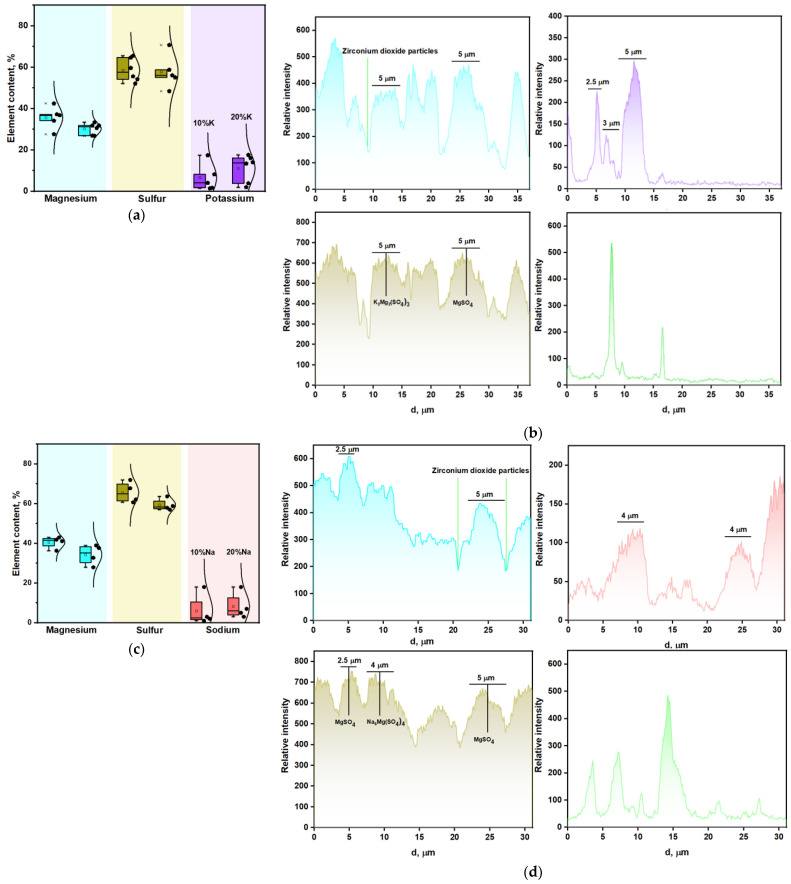
(**a**,**c**) Element content in Mg10KY-TZP and Mg20KY-TZP, and Mg10NaY-TZP and Mg20NaY-TZP samples according to EDX; (**b**,**d**) elemental line scans of the Mg10KY-TZP and Mg10NaY-TZP samples.

The elemental line scans of magnesium, potassium, sulfur, and zirconia are presented in Figure 5. It was demonstrated that sulfur peaks were mostly followed by magnesium peaks, outlining the grains revealed by the SEM images. This means that magnesium sulfate was in a dominant phase in most of the grains. The average size of these grains was 5–7 μm according to the peak width in the spectra. The above observations were of a group of magnesium sulfate grains that were not overlapped with K-containing grains, as observed in the SEM images. The grain boundaries, as well as pores, could be easily detected from line scans using drops of the spectra intensity. Several pores were filled by aggregates of zirconium dioxide nanoparticles with diameters of less than 1 μm.

According to the line scans, the average size of the grains in the MgSO_4_-K_2_SO_4_ system was lower than in the MgSO_4_-Na_2_SO_4_ system. For MgSO_4_-K_2_SO_4_ it was 3–4 μm, while for the MgSO_4_-Na_2_SO_4_ system it was 4–5 μm. Such an estimation was made from the average size of all of the grains presented. Potassium and sodium elemental line scans provided some information on the distribution of K-containing (K_2_Mg_2_(SO_4_)_3_) and Na-containing (Na_6_Mg(SO_4_)_4_) grains. Thereby, the combination of line scanning and BSE/SE imaging provided enough information about the actual grain sizes and the spatial distribution of the grains in the studied ceramics. Na-containing grains consisted of two different types: small, with an average size of 1–2 μm, and large, with an average size of 5–7 μm. K-containing grains were not widely presented in the studied line scans; however, they tended to differentiate similar to the 1 μm and 4–5 μm grains. It should also be mentioned that some faint peaks in the line scans stemmed from the surface topography.

The magnesium to sodium or potassium ratios in the obtained samples according to EDX are presented in Table 2. The calculated ratios of Mg/Na (Mg10Na—5, Mg20Na—2.5) and Mg/K (Mg10K—5, Mg20K—2.5) correspond to those found for Mg10K—5.3 and Mg20K—2.7. For the sodium-containing samples, the obtained ratios were slightly higher: Mg10Na—6.5 and Mg20Na—4.1. This was probably due to the uneven distribution (Na-containing samples also consisted of larger grains) of the element with a lower content throughout the sample.

The same trends were observed for the Na-containing ceramics. The melting point of sodium sulfate was closer to the sintering temperature in comparison with potassium sulfate. Such difference led to faster grain growth in the samples with a higher content of sodium. According to the elemental line scans, Na-containing grains (most likely Na_6_Mg(SO_4_)_4_ phase) had an average diameter of 6–7 μm, and were larger than the K-containing grains by 2–3 μm, as expected. Another essential feature was the influence of zirconia particles on grain growth. ZrO_2_ nanoparticles had an average size of 20–40 nm (Figure 3 and Figure S2) and formed aggregates that had a size of up to 1 μm. Apparently, they inhibit grain growth. This effect was observed in both SEM images and line scans. For instance, near the locations of the intensive peaks (large agglomerates) of zirconia in the line scans, the sodium peaks were narrower than in the rest of the length of the corresponding line scan (Figure 5).

**Figure 6:**
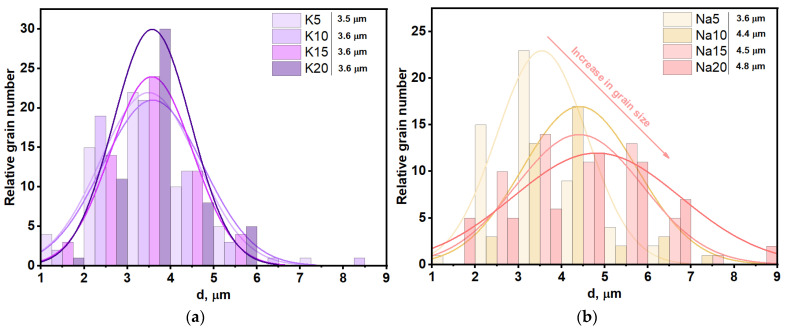
Grain size distribution in: (**a**) K-containing, (**b**) Na-containing ceramic samples.

The grain size distribution is presented in Figure 6.

The grain sizes were also calculated from the SEM images using ImageJ software. It is worth noting in the linescans, that in the Na-containing samples, grains tended to grow with an increase in the amount of sodium, but not excessively (up to 33%). Nonetheless, this effect was not observed for potassium-containing samples, e.g., the difference between the average grain sizes for Mg5K and Mg20K was less than 1%.

**Figure 7:**
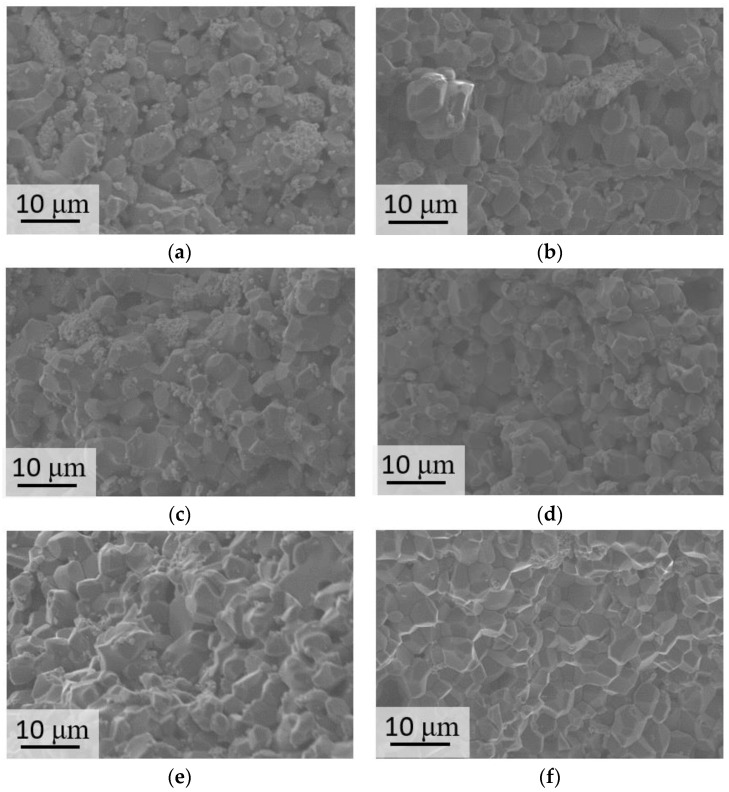

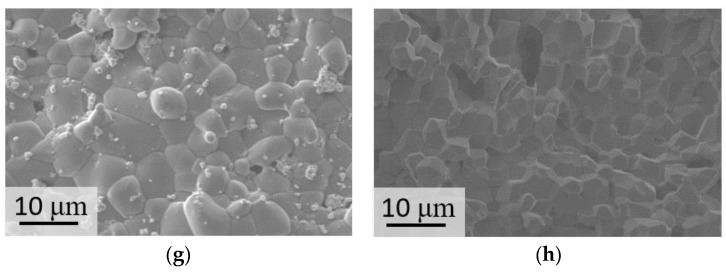
SEM images (SE2-type) of the samples: (**a**) Mg5NaY-TZP, (**b**) Mg5KY-TZP, (**c**) Mg10NaY-TZP, (**d**) Mg10KY-TZP, (**e**) Mg15NaY-TZP, (**f**) Mg15KY-TZP, (**g**) Mg20NaY-TZP, (**h**) Mg20KY-TZP.

SEM images of the sample surface are presented at Figure 7 and Figure S3.

**Figure 8:**
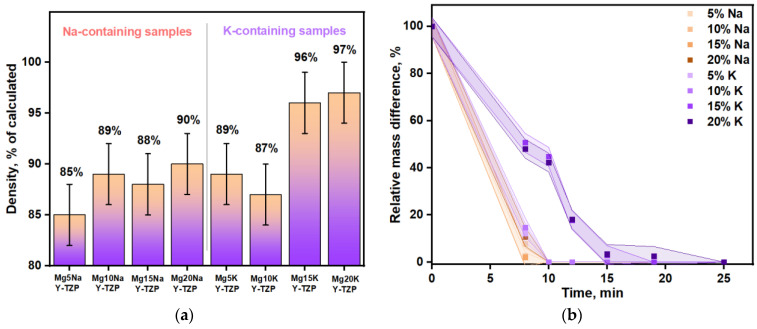
(**a**) Bulk density data; (**b**) mass change during the dissolution process.

The lowest density was obtained for the sample contained 5% sodium sulfate (85%). The further addition of sodium sulfate led to an increase in density up to 90% (Figure 8a) due to restrained grain growth and the removal of pores. Such a tendency could also be traced for the Mg10Na, Mg15Na, and Mg20Na samples. The corresponding SEM images (Figure 7a–d) could verify these conclusions. The Mg5Na sample was slightly porous and the grains were not in a very tight contact. Here, the addition of 10% and 15% sodium caused minor grain growth and led to densification of the samples, because of the higher content of the more fusible (compared with MgSO_4_) Na_6_Mg(SO_4_)_4_ phase. The densest sample with a notable lack of pores and the largest grains (4,6 μm average, a lot of grains had diameters up to 7 μm) was Mg20Na. Furthermore, K-containing samples demonstrated a higher density than the Na-containing samples (Figure 8a). The samples with a low content (5–10%) of potassium tended to have a density up to 90%, being close to that of the Na-containing samples. K-containing samples also had an ascending trend in density with an increase in the potassium content up to 20%. These samples had a sufficiently high density up to 98% (96% on average for Mg15K and 97% on average for Mg20K). The grain size increment between the least dense and densest samples was 5% for sodium-containing ceramics and 10% for potassium-containing ceramics. This difference originated from the different contribution of the grain growth to the densification process for sodium- and potassium-containing samples. As it was already noted, the grain size increment between the 5% sodium and 20% sodium samples was 33%, while for the potassium-containing samples it was less than 1%. Thereby, densification occurred significantly in the potassium-containing ceramics, apparently due to the elimination of pores.

Another important feature is the inhibition of grain growth in the ceramic composites by zirconia nanoparticles. The difference between grain sizes in Mg10Na, Mg15Na, and Mg20Na was not significant, despite the increase in the sodium content and the tendency to recrystallization. In potassium-containing samples, this tendency was also observed in the samples prepared without zirconia. Hence, the addition of zirconia particles did not significantly inhibit grain growth and promoted densification.

As the studied materials were intended for use as soluble sacrificial molds, another important characteristic is their dissolution time (Figure 8b). To conduct this experiment, samples with the same mass (0.1 g) were dissolved in distilled water. The acquired dissolution time was expected to be significantly correlated with the sample density. The lowest dissolution time (~8 min) was found for Mg5Na, the least dense sample (85%). Complete dissolution time for all of the other samples with a similar density (87–90%) was 10 min, except for the Mg15K and Mg20K samples. As for the densest samples with a density of 96–97%, the dissolution time was 50–150% longer and reached 15 min for Mg15K and 25 min for Mg20K. Thus, the obtained dissolving times were not long enough, so there were no solubility limits for these samples in terms of their use as soluble “sacrificial” molds. Furthermore, zirconium dioxide particles did not interfere with the dissolution and could be completely removed during the dissolution of the sulfate ceramics. Therefore, they did not remain in the casted polymer composite.

**Figure 9:**
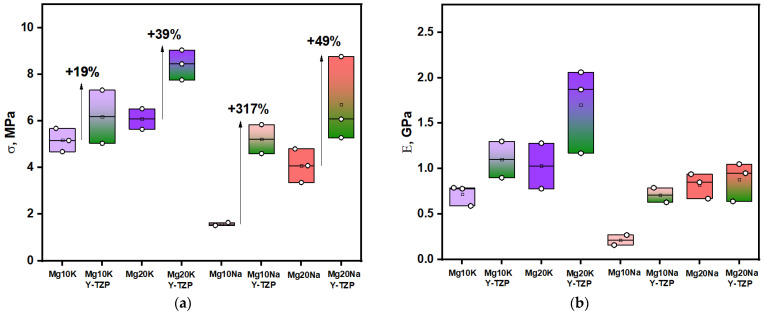
(**a**) Compressive strength of the ceramic samples; (**b**) Young’s modulus of the ceramic samples.

The strength and Young’s modulus of the obtained ceramics are presented in Figure 9. They had the same trends as the density.

The best strength and rigidity were obtained for Mg20KY-TZP, which was the densest material produced. The Mg20KY-TZP sample had slightly more tensile strength than Mg10KY-TZP and all of the Na-containing samples. The influence of zirconia on the tensile strength and Young’s modulus was significant for all of the samples: from 19% for the Mg10K line to 49% for the Mg20Na line. In sodium-containing samples, the strengthening effect was more significant, as the phase distribution in the samples without zirconia was highly irregular, especially for the Mg10Na sample, which had the lowest tensile strength and Young’s modulus.

Initially, the precursor samples, obtained after milling, contained monoclinic zirconia. The latter could still inhibit grain growth and crack propagation, but transformation toughening could not be implemented. Thereby, the addition of the tetragonal zirconia powder was suggested to increase the toughening of the final ceramics. Transformation toughening (T→M) is sensitive to mechanical and thermal stresses, and is a source of toughening in Y-stabilized zirconia [58]. Tetragonal phase is thermodynamically stable until 1367 K [59]; so, under sintering conditions, T→M transformation is not likely to occur [60,61]. Another significant feature is that 3 wt.% of yttrium oxide is optimal for tetragonal phase stabilizing; otherwise, with a higher amount of Y_2_O_3_ (7 wt.%), the mechanical characteristics tended to worsen [62,63]. In the current research, we proposed adding 5 wt.% Y-TZP to the raw mixture of sulfates. This was enough to ensure transformational toughening and grain growth deceleration. Nevertheless, the amount of the zirconia that was added was not limited by 5 wt.% and its influence on the mechanical characteristics of various ceramic composites is an interesting object for the further studies. The increase in hardness could possibly be obtained in a similar way as for the zirconia tetragonal alumina composites (ZTA) [64,65]. Effective toughening could also shift the application of such materials to the processes of medium pressure injection molding (MPIM) [66] and high-pressure injection molding (HPIM) [67].

**Figure 10:**
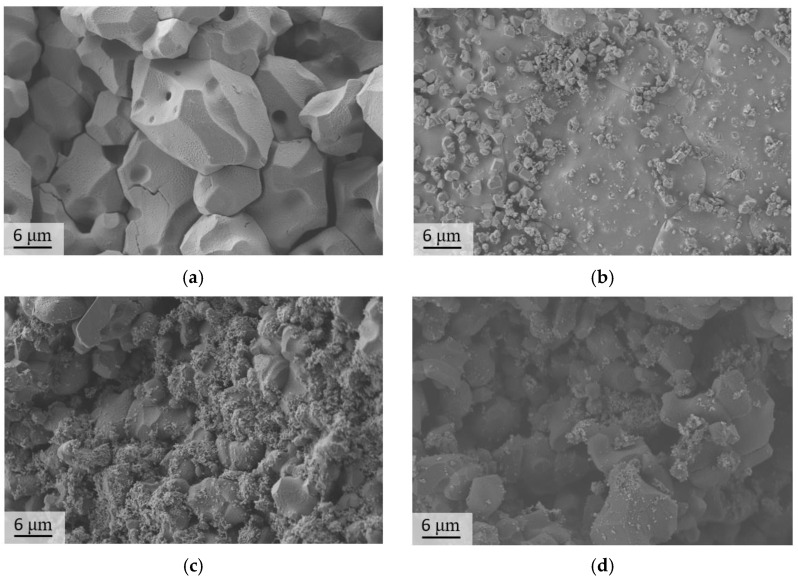
SEM images (SE2) of the fractured samples: (**a**) Mg20K, (**b**) Mg10K, (**c**) Mg20KY-TZP, and (**d**) Mg10KY-TZP.

The specifics of the microstructure of zirconia-containing and non-containing samples can also be clearly demonstrated in the SEM images of the surface of the samples after mechanical testing (Figure 10).

The zirconia-containing samples had a lower grain size (up to 6 μm) and relatively higher density (up to 98% for Mg20KY-TZP). There were no sufficient cracks observed in the SEM images; thereby, crack propagation tended to develop along the grain boundaries. The observations confirmed the necessity of the addition of zirconia nanoparticles in order to obtain sulfate ceramic/zirconia composites. The samples without zirconia also had considerable density (up to 95% for Mg20K); nonetheless, worse mechanical characteristics were demonstrated by these samples. Moreover, the SEM images demonstrated some cracks spread inside the grains (transgranular cracks).

**Figure 11:**
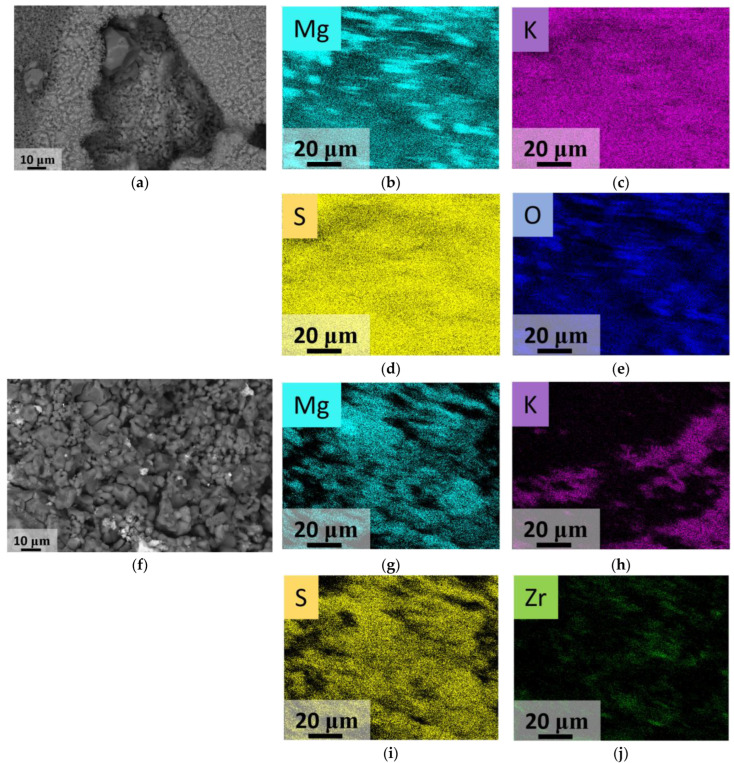
(**a**,**f**) SEM images (SE2) of the fractured Mg10K and Mg10KY-TZP samples; (**b**–**e**,**g**–**j**) corresponding elemental maps.

Several features of the microstructure of zirconia-containing and non-containing samples can also be demonstrated at the SEM images and elemental maps of the surface of the samples after mechanical testing (Figure 11).

The elemental maps confirmed that Mg10K had large K-containing grains with small separated MgSO_4_ grains between them. They also tended to concentrate in the pores between the K_2_Mg_2_(SO_4_)_3_ grains. The addition of zirconia particles led to the formation of smaller grains in the K_2_Mg_2_(SO_4_)_3_ phase. Furthermore, a more uniform distribution of the magnesium grains was achieved, as the grains of magnesium sulfate and mixed magnesium−potassium sulfate were close in size in comparison to the samples without zirconia additives. Moreover, the zirconia nanoparticles were distributed evenly in the ceramics that were obtained.

**Figure 12:**
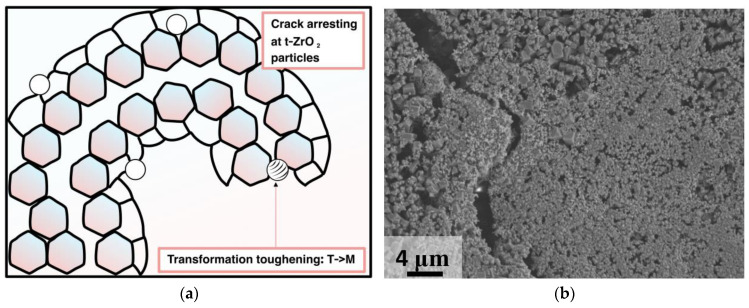
(**a**) Scheme of crack arresting by the ZrO_2_ particle; (**b**) SEM image of the fractured Mg20KY-TZP sample.

Crack propagation reflects toughening mechanisms in ceramics. In polycrystalline porous ceramics, the arrest of cracks by pores, crack reorientation, and branching are typical mechanisms of toughening [68]. The addition of zirconia particles induced the formation of pseudoplastic zones around the stressed inclusions, leading to the additional deceleration of the crack opening (Figure 12). In the SEM image of the Mg20KY-TZP sample, the crack initiated by fracturing the sample during mechanical testing with a width of 3–4 μm is presented. In the highlighted zone (above bright spot of zirconia agglomerate), which is related to the higher concentration of zirconia nanoparticles, the crack became curved and almost disappeared.

**Figure 13:**
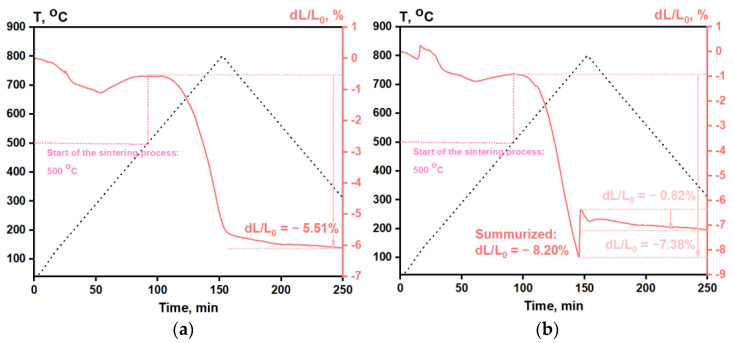
Shrinkage curves for (**a**) Mg20K and (**b**) Mg20KY-TZP.

Besides fracture toughening, Y-TZP nanoparticles can inhibit grain growth. Such an effect was shown for the potassium-containing samples without the addition of zirconia (Figure 13a) and as well as with the addition (Figure 13b).

According to the dilatometry, the sintering process started at the same temperature for Mg20K and Mg20KY-TZP, but the final dL/L_0_ value for the zirconia-containing sample was −8.2%, while for the Mg20K sample the value dL/L_0_ was equal to −5.51%. The rapid growth in the dL/L_0_ for K20Y-TZP sample in the interval of 140–150 min occurred as a result of minor cracking of the sample. The better densification could be explained by the slower grain growth due to the presence of zirconia nanoparticles. For the sodium-containing samples, this process was more complicated, as the sintering temperature was much closer to the melting point of the mixed sulfate phase. According to the TG (Figure S4) for the Mg20KY-TZP sample, two endo peaks were observed at temperatures of 575 and 750 °C. They correspond to the eutectic temperatures in the corresponding phase diagram [48]. The peak at 750 °C was very broad and asymmetrical with a cut-off at 752 °C, which could be regarded as corresponding to the solid-state reaction between K_2_SO_4_ and MgSO_4_, leading to the formation of K_2_Mg_2_(SO_4_)_3._

**Figure 14:**
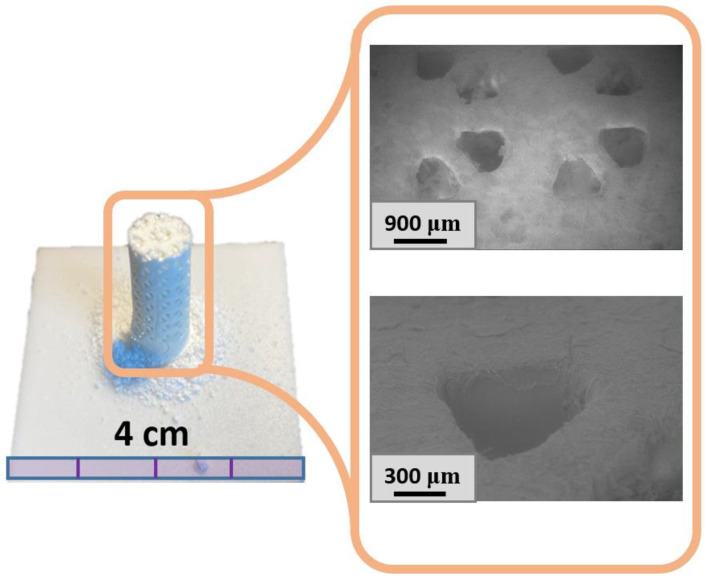
Fabricated sulfate ceramic (Mg20KY-TZP) mold.

The resulting 3D molds are demonstrated in Figure 14.

The achieved resolution was obviously better than 50 µm. Moreover, there were no voids and discontinuities in the samples associated with the press-filtration, which caused uneven filling of the 3D-printed mold. Fabricated macroporous ceramics can be implemented as sacrificial molds for low-pressure injection molding to produce bioresorbable thermoplastic polymer/calcium phosphate composites for bone tissue regeneration.

## 4. Conclusions

In this work, a novel sacrificial sulfate ceramic material for application using a low-pressure injection molding technique was proposed. Magnesium sulfate with the addition of 5–20% sodium sulfate and magnesium sulfate with the addition of 5–20% potassium sulfate were used as precursors. EDX data confirmed that the expected ratios of Mg/Na (MgSO_4_/Na_2_SO_4_ = 91/9%—5 and MgSO_4_/Na_2_SO_4_ = 83/17%—2.5) and Mg/K (MgSO_4_/K_2_SO_4_ = 91/9%—5 and MgSO_4_/K_2_SO_4_ = 83/17%—2.5) corresponded to what was obtained for (MgSO_4_/K_2_SO_4_ = 91/9%—5.3 and MgSO_4_/K_2_SO_4_ = 83/17%—2.7) and slightly lower than for (MgSO_4_/Na_2_SO_4_ = 91/9%—6.5 and MgSO_4_/Na_2_SO_4_ = 83/17%—4.1). The main phases formed in the MgSO_4_-Na_2_SO_4_ mixture (T_sintering_ = 750 °C) were MgSO_4_ and Na_6_Mg(SO_4_)_4_, while in the MgSO_4_-K_2_SO_4_ mixture (T_sintering_ = 800 °C), MgSO_4_ and K_2_Mg_2_(SO_4_)_3_ (Langbeinite) formed. It was also shown that the addition of 5% of the tetragonal zirconia increased the compressive strength by 39% for the MgSO_4_/K_2_SO_4_ = 83/17% sample, and 49% for the MgSO_4_/Na_2_SO_4_ = 83/17% sample. The maximum compressive strength was achieved for the (MgSO_4_/K_2_SO_4_ = 83/17% sample—8.4 ± 0.6 MPa. Moreover, the complete dissolution time for all of the samples did not exceed 25 min. Using stereolithographic 3D printing, inverted macroporous sulfate ceramic molds were produced. They were implemented to biocompatible composite bone tissue implant prototype formation through low pressure injection molding. The strength values of the obtained materials will allow sulfate ceramics to be used in low-pressure injection molding for such prototypes.

## Figures and Tables

**Table 1:** Sample abbreviations and phase compositions.

Label	Phase Composition, mol.%		
	MgSO_4_	Na_2_SO_4_	K_2_SO_4_
Mg5Na and Mg5NaY-TZP	95	5	–
Mg10Na and Mg10NaY-TZP	91	9	–
Mg15Na and Mg15NaY-TZP	87	13	–
Mg20Na and Mg20NaY-TZP	83	17	–
Mg5K and Mg5KY-TZP	95	–	5
Mg10K and Mg10KY-TZP	91	–	9
Mg15K and Mg15KY-TZP	87	–	13
Mg20K and Mg20KY-TZP	83	–	17

**Table 2:** Mg/(Na, K) ratios according to EDX in comparison with the calculated values.

Sample	Calculated Mg/(Na, K) Ratio	Found Mg/(Na, K) Ratio
Mg10Na	5	6.5
Mg20Na	2.5	4.1
Mg10K	5	5.3
Mg20K	2.5	2.7

## Data Availability

Not applicable.

## Supplementary Materials

The following supporting information can be downloaded at: https://www.mdpi.com/article/10.3390/ma16083077/s1. Figure S1: TG/DTA for magnesium sulfate. Figure S2: SEM images of the zirconia nanoparticles: (**a**) BSE detector and (**b**) SE2 detector. Figure S3: SEM images of the obtained ceramics. Figure S4: TG/DTA data for Mg20KY-TZP.

## References

[1] Juhasz J.A., Best S.M. (2012). Bioactive Ceramics: Processing, Structures and Properties. J. Mater. Sci..

[2] Navarro M., Michiardi A., Castaño O., Planell J.A. (2008). Biomaterials in Orthopaedics. J. R. Soc. Interface.

[3] Neumann M., Epple M. (2006). Composites of Calcium Phosphate and Polymers as Bone Substitution Materials. Eur. J. Trauma.

[4] Valtanen R.S., Yang Y.P., Gurtner G.C., Maloney W.J., Lowenberg D.W. (2021). Synthetic and Bone Tissue Engineering Graft Substitutes: What Is the Future?. Injury.

[5] Kazakova G., Safronova T., Golubchikov D., Shevtsova O., Rau J.V. (2021). Resorbable Mg^2+^—Containing Phosphates for Bone Tissue Repair. Materials.

[6] Wang N., Thameem Dheen S., Fuh J.Y.H., Senthil Kumar A. (2021). A Review of Multi-Functional Ceramic Nanoparticles in 3D Printed Bone Tissue Engineering. Bioprinting.

[7] Kamboj N., Ressler A., Hussainova I. (2021). Bioactive Ceramic Scaffolds for Bone Tissue Engineering by Powder Bed Selective Laser Processing: A Review. Materials.

[8] Marques A., Miranda G., Silva F., Pinto P., Carvalho Ó. (2020). Review on Current Limits and Potentialities of Technologies for Biomedical Ceramic Scaffolds Production. J. Biomed. Mater. Res. Part B Appl. Biomater..

[9] Samavedi S., Whittington A.R., Goldstein A.S. (2013). Calcium Phosphate Ceramics in Bone Tissue Engineering: A Review of Properties and Their Influence on Cell Behavior. Acta Biomater..

[10] Evdokimov P.V., Tikhonova S.A., Kiseleva A.K., Filippov Y.Y., Novoseletskaya E.S., Efimenko A.Y., Putlayev V.I. (2021). Effect of the Pore Size on the Biological Activity of β-Ca3(PO4)2-Based Resorbable Macroporous Ceramic Materials Obtained by Photopolymerization. Russ. J. Inorg. Chem..

[11] Tikhonov A., Putlayev V. (2022). Synthesis and Thermal Behaviour of Calcium Alkyl Phosphates as Bioceramic Precursors. Ceramics.

[12] Kumar A., Mir S.M., Aldulijan I., Mahajan A., Anwar A., Leon C.H., Terracciano A., Zhao X., Su T.L., Kalyon D.M. (2020). Load-Bearing Biodegradable PCL-PGA-Beta TCP Scaffolds for Bone Tissue Regeneration. J. Biomed. Mater. Res. Part B Appl. Biomater..

[13] Zhukova P.A., Senatov F.S., Zadorozhnyy M.Y., Chmelyuk N.S., Zaharova V.A. (2021). Polymer Composite Materials Based on Polylactide with a Shape Memory Effect for “Self-Fitting” Bone Implants. Polymers.

[14] Evans K.A., Kennedy Z.C., Arey B.W., Christ J.F., Schaef H.T., Nune S.K., Erikson R.L. (2018). Chemically Active, Porous 3D-Printed Thermoplastic Composites. ACS Appl. Mater. Interfaces.

[15] Golubchikov D., Safronova T.V., Nemygina E., Shatalova T.B., Tikhomirova I.N., Roslyakov I.V., Khayrutdinova D., Platonov V., Boytsova O., Kaimonov M. (2023). Powder Synthesized from Aqueous Solution of Calcium Nitrate and Mixed-Anionic Solution of Orthophosphate and Silicate Anions for Bioceramics Production. Coatings.

[16] Bernard M., Jubeli E., Pungente M.D., Yagoubi N. (2018). Biocompatibility of Polymer-Based Biomaterials and Medical Devices-Regulations, In Vitro Screening and Risk-Management. Biomater. Sci..

[17] Williams D.F. (2008). On the Mechanisms of Biocompatibility. Biomaterials.

[18] Zuev D.M., Golubchikov D.O., Evdokimov P.V., Putlyaev V.I. (2022). Synthesis of Amorphous Calcium Phosphate Powders for Production of Bioceramics and Composites by 3D Printing. Russ. J. Inorg. Chem..

[19] Hurle K., Oliveira J.M., Reis R.L., Pina S., Goetz-Neunhoeffer F. (2021). Ion-Doped Brushite Cements for Bone Regeneration. Acta Biomater..

[20] Spirandeli B.R., Ribas R.G., Amaral S.S., Martins E.F., Esposito E., Vasconcellos L.M.R., Campos T.M.B., Thim G.P., Trichês E.S. (2021). Incorporation of 45S5 Bioglass via Sol-Gel in β-TCP Scaffolds: Bioactivity and Antimicrobial Activity Evaluation. Mater. Sci. Eng. C.

[21] Hutmacher D.W. (2000). Scafflds in Tissue Engineering Bone and Cartilage. Biomaterials.

[22] Rezvani Ghomi E., Nourbakhsh N., Akbari Kenari M., Zare M., Ramakrishna S. (2021). Collagen-Based Biomaterials for Biomedical Applications. J. Biomed. Mater. Res. Part B Appl. Biomater..

[23] Jin S., Xia X., Huang J., Yuan C., Zuo Y., Li Y., Li J. (2021). Recent Advances in PLGA-Based Biomaterials for Bone Tissue Regeneration. Acta Biomater..

[24] Schilling A.F., Filke S., Brink S., Korbmacher H., Amling M., Rueger J.M. (2006). Osteoclasts and Biomaterials. Eur. J. Trauma.

[25] Karageorgiou V., Kaplan D. (2005). Porosity of 3D Biomaterial Scaffolds and Osteogenesis. Biomaterials.

[26] Murphy C.M., Haugh M.G., O’Brien F.J. (2010). The Effect of Mean Pore Size on Cell Attachment, Proliferation and Migration in Collagen-Glycosaminoglycan Scaffolds for Bone Tissue Engineering. Biomaterials.

[27] Goto M., Matsumine A., Yamaguchi S., Takahashi H., Akeda K., Nakamura T., Asanuma K., Matsushita T., Kokubo T., Sudo A. (2020). Osteoconductivity of Bioactive Ti-6Al-4V Implants with Lattice-Shaped Interconnected Large Pores Fabricated by Electron Beam Melting. J. Biomater. Appl..

[28] Shuai C., Yang W., Feng P., Peng S., Pan H. (2021). Accelerated Degradation of HAP/PLLA Bone Scaffold by PGA Blending Facilitates Bioactivity and Osteoconductivity. Bioact. Mater..

[29] Sadeghian A., Kharaziha M., Khoroushi M. (2022). Osteoconductive Visible Light-Crosslinkable Nanocomposite for Hard Tissue Engineering. Colloids Surf. A Physicochem. Eng. Asp..

[30] Sahithi K., Swetha M., Ramasamy K., Srinivasan N., Selvamurugan N. (2010). Polymeric Composites Containing Carbon Nanotubes for Bone Tissue Engineering. Int. J. Biol. Macromol..

[31] Pietrzykowska E., Romelczyk-Baishya B., Chodara A., Koltsov I., Smogór H., Mizeracki J., Pakieła Z., Łojkowski W. (2022). Microstructure and Mechanical Properties of Inverse Nanocomposite Made from Polylactide and Hydroxyapatite Nanoparticles. Materials.

[32] Beatrice C.A.G., Shimomura K.M.B., Backes E.H., Harb S.V., Costa L.C., Passador F.R., Pessan L.A. (2020). Engineering Printable Composites of Poly (ε-Polycaprolactone)/β-Tricalcium Phosphate for Biomedical Applications. Polym. Compos..

[33] Rabiei M., Raziyan M.S., Ebrahimi-Kahrizsangi R., Nasiri S., Palevicius A., Janusas G., Vilkauskas A. (2022). Effects of 5 Wt.% Polycaprolactone, Polyhydroxybutyrate and Polyvinyltrimethoxysilane on the Properties of Ag/Zn/Mg Alloy. Materials.

[34] Tayebi M., Parham S., Abbastabbar Ahangar H., Zargar Kharazi A. (2021). Preparation and Evaluation of Bioactive Bilayer Composite Membrane PHB/β-TCP with Ciprofloxacin and Vitamin D3 Delivery for Regenerative Damaged Tissue in Periodontal Disease. J. Appl. Polym. Sci..

[35] Barczewski M., Mysiukiewicz O., Matykiewicz D., Skórczewska K., Lewandowski K., Andrzejewski J., Piasecki A. (2020). Development of Polylactide Composites with Improved Thermomechanical Properties by Simultaneous Use of Basalt Powder and a Nucleating Agent. Polym. Compos..

[36] Ghayor C., Weber F.E. (2018). Osteoconductive Microarchitecture of Bone Substitutes for Bone Regeneration Revisited. Front. Physiol..

[37] Ligon S.C., Liska R., Stampfl J., Gurr M., Mülhaupt R. (2017). Polymers for 3D Printing and Customized Additive Manufacturing. Chem. Rev..

[38] Helú M.A.B., Liu L. (2021). Fused Deposition Modeling (FDM) Based 3D Printing of Microelectrodes and Multi-Electrode Probes. Electrochim. Acta.

[39] Silva A.D.R., Rigoli W.R., Osiro D., Mello D.C.R., Vasconcellos L.M.R., Lobo A.O., Pallone E.M.J.A. (2018). Surface Modification Using the Biomimetic Method in Alumina-Zirconia Porous Ceramics Obtained by the Replica Method. J. Biomed. Mater. Res. Part B Appl. Biomater..

[40] Liska R., Schwager F., Maier C., Cano-Vives R., Stampfl J. (2005). Water-Soluble Photopolymers for Rapid Prototyping of Cellular Materials. J. Appl. Polym. Sci..

[41] Xie L., Yu H., Deng Y., Yang W., Liao L., Long Q. (2016). Preparation, Characterization and in Vitro Dissolution Behavior of Porous Biphasic α/β-Tricalcium Phosphate Bioceramics. Mater. Sci. Eng. C.

[42] Safronova T.V., Shatalova T.B., Filippov Y.Y., Toshev O.U., Knotko A.V., Vaimugin L.A., Savchenkova D.V. (2022). Na_2_O-CaO-SO_3_ Ceramics as Promising Inorganic Porogens. Glass Ceramics..

[43] Cui P., Wu C.-R., Chen J., Luo F.-M., Kou S.-C. (2021). Preparation of Magnesium Oxysulfate Cement as a 3D Printing Material. Constr. Build. Mater..

[44] Li G., Tang S., Yang L., Qian L., Liu F., Fan Z., Zuo K., Wei Q., Jiang W. (2019). Fabrication of Soluble Salt-Based Support for Suspended Ceramic Structure by Layered Extrusion Forming Method. Mater. Des..

[45] Li W., Ghazanfari A., McMillen D., Leu M.C., Hilmas G.E., Watts J. (2017). Fabricating Ceramic Components with Water Dissolvable Support Structures by the Ceramic On-Demand Extrusion Process. CIRP Ann.-Manuf. Technol..

[46] Martínez-Vázquez F.J., Pajares A., Miranda P. (2018). A Simple Graphite-Based Support Material for Robocasting of Ceramic Parts. J. Eur. Ceram. Soc..

[47] Rowe J.J., Morey G.W., Smber C.C. (1967). The ternary system K2SO4-MgSO4-CaSO4. J. Inorg. Nucl. Chem..

[48] Du H. (2000). Thermodynamic Assesment of the K_2_SO_4_-Na_2_SO_4_-MgSO_4_-CaSO_4_ System. J. Phase Equilibria Diffus..

[49] Körber S., Völkl R., Glatzel U. (2021). 3D printed polymer positive models for the investment casting of extremely thin-walled single crystals. J. Mater. Process. Technol..

[50] Oosterbeek R.N., Margaronis K.I., Zhang X.C., Best S.M., Cameron R.E. (2021). Non-Linear Dissolution Mechanisms of Sodium Calcium Phosphate Glasses as a Function of PH in Various Aqueous Media. J. Eur. Ceram. Soc..

[51] Huang J., Sun Y., Zhang Y., Zou G., Yan C., Cong S., Lei T., Dai X., Guo J., Lu R. (2018). A New Member of Electrocatalysts Based on Nickel Metaphosphate Nanocrystals for Efficient Water Oxidation. Adv. Mater..

[52] Zhang X., Liu C., Jiang M. (2021). Effect of Na Ions on Melt Structure and Viscosity of CaO-SiO_2_-Na_2_O by Molecular Dynamics Simulations. ISIJ Int..

[53] German R.M., Suri P., Park S.J. (2009). Review: Liquid Phase Sintering. J. Mater. Sci..

[54] Qi L., He S., Chen C., Jiang B., Hao Y., Ye H., Yang R., Du K. (2020). Diffusional-Displacive Transformation in a Metastable β Titanium Alloy and Its Strengthening Effect. Acta Mater..

[55] Chen J., Zhou L., Liang J., Liu B., Liu J., Chen R., Deng X., Wu S., Huang M. (2021). Effect of Initial WC Particle Size on Grain Growth Behavior and Gradient Structure Formation of Bilayer Functionally Graded Cemented Carbides. Mater. Chem. Phys..

[56] Gad M.M., Abualsaud R., Rahoma A., Al-Thobity A.M., Al-Abidi K.S., Akhtar S. (2018). Effect of Zirconium Oxide Nanoparticles Addition on the Optical and Tensile Properties of Polymethyl Methacrylate Denture Base Material. Int. J. Nanomed..

[57] Kim M.I., Kim S., Kim T., Lee D.K., Seo B., Lim C.S. (2017). Mechanical and Thermal Properties of Epoxy Composites Containing Zirconium Oxide Impregnated Halloysite Nanotubes. Coatings.

[58] Mamivand M., Zaeem M.A., Kadiri H.E., Chen L.Q. (2013). Phase Field Modeling of the Tetragonal-to-Monoclinic Phase Transformation in Zirconia. Acta Mater..

[59] Wang C., Zinkevich M., Aldinger F. (2006). The Zirconia-Hafnia System: DTA Measurements and Thermodynamic Calculations. J. Am. Ceram. Soc..

[60] Navrotsky A. (2005). Thermochemical Insights into Refractory Ceramic Materials Based on Oxides with Large Tetravalent Cations. J. Mater. Chem..

[61] Gehensel R.J., Zierold R., Schaan G., Shang G., Petrov A.Y., Eich M., Blick R., Krekeler T., Janssen R., Furlan K.P. (2021). Improved Thermal Stability of Zirconia Macroporous Structures via Homogeneous Aluminum Oxide Doping and Nanostructuring Using Atomic Layer Deposition. J. Eur. Ceram. Soc..

[62] Jang B.K., Lee J.H., Fisher C.A.J. (2021). Mechanical Properties and Phase-Transformation Behavior of Carbon Nanotube-Reinforced Yttria-Stabilized Zirconia Composites. Ceram. Int..

[63] Magnani G., Fabbri P., Leoni E., Salernitano E., Mazzanti F. (2021). New Perspectives on Zirconia Composites as Biomaterials. J. Compos. Sci..

[64] Bergamo E.T.P., Cardoso K.B., Lino L.F.O., Campos T.M.B., Monteiro K.N., Cesar P.F., Genova L.A., Thim G.P., Coelho P.G., Bonfante E.A. (2021). Alumina-Toughened Zirconia for Dental Applications: Physicochemical, Mechanical, Optical, and Residual Stress Characterization after Artificial Aging. J. Biomed. Mater. Res. Part B Appl. Biomater..

[65] Chłędowska J., Wyrwa J., Rękas M., Brylewski T. (2022). Effects of Aluminum Oxide Addition on Electrical and Mechanical Properties of 3 Mol% Yttria-Stabilized Tetragonal Zirconia Electrolyte for IT-SOFCs. Materials.

[66] Jewad R., Bentham C., Hancock B., Bonfield W., Best S.M. (2008). Dispersant Selection for Aqueous Medium Pressure Injection Moulding of Anhydrous Dicalcium Phosphate. J. Eur. Ceram. Soc..

[67] Wang Z.G., Hsiao B.S., Stribeck N., Gehrke R. (2002). Nanostructure Evolution of Isotropic High-Pressure Injection-Molded UHMWPE during Heating. Macromolecules.

[68] Bai R., Sun Q., He Y., Peng L., Zhang Y., Zhang L., Lu W., Deng J., Zhuang Z., Yu T. (2022). Ceramic Toughening Strategies for Biomedical Applications. Front. Bioeng. Biotechnol..

