# Supplementary material for: Three-Dimensional-Printed Molds from Water-Soluble Sulfate Ceramics for Biocomposite Formation through Low-Pressure Injection Molding
Source: Materials (Basel). 2023 Apr 13;16(8):3077. doi: 10.3390/ma16083077 (PMC10145792; doi:10.3390/ma16083077)
Supplement: Supplementary file 1 [file materials-16-03077-s001.zip › materials-2303067-supplementary.pdf]

Supplementary materials

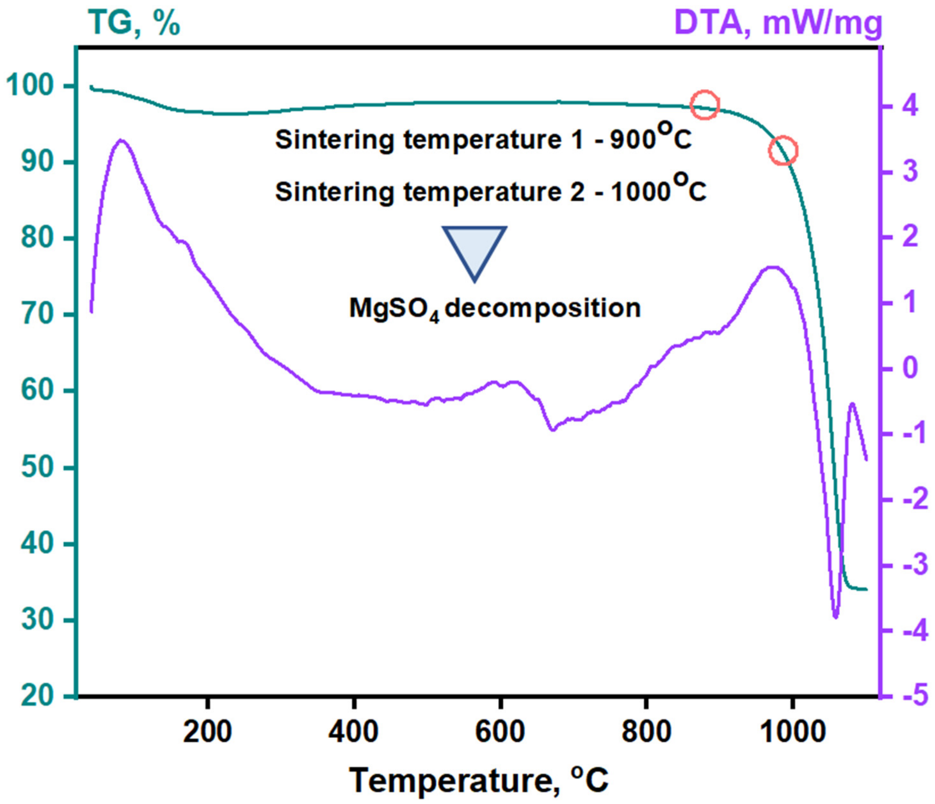

Figure S1. TG/DTA for magnesium sulfate.

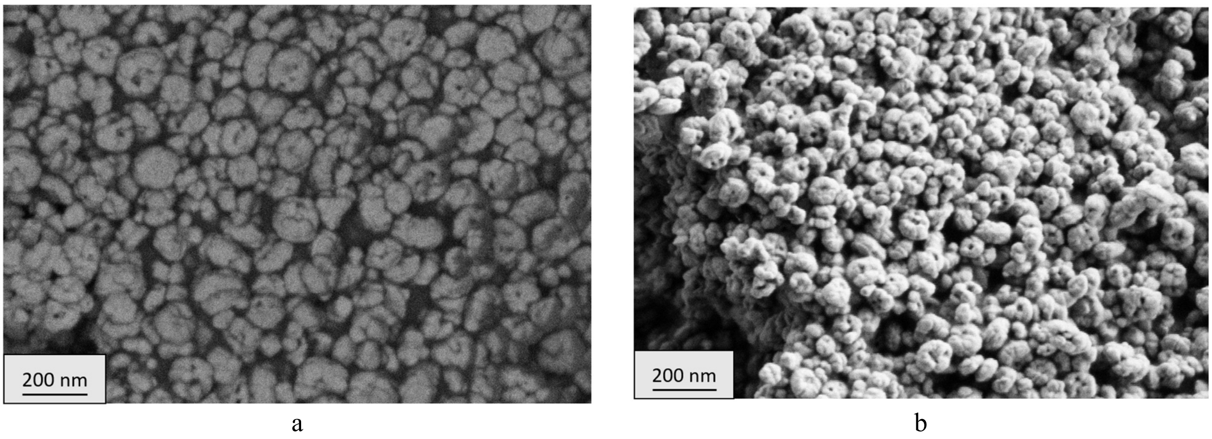

Figure S2. SEM images of the zirconia nanoparticles: (a) BSE detector and (b) SE2 detector.

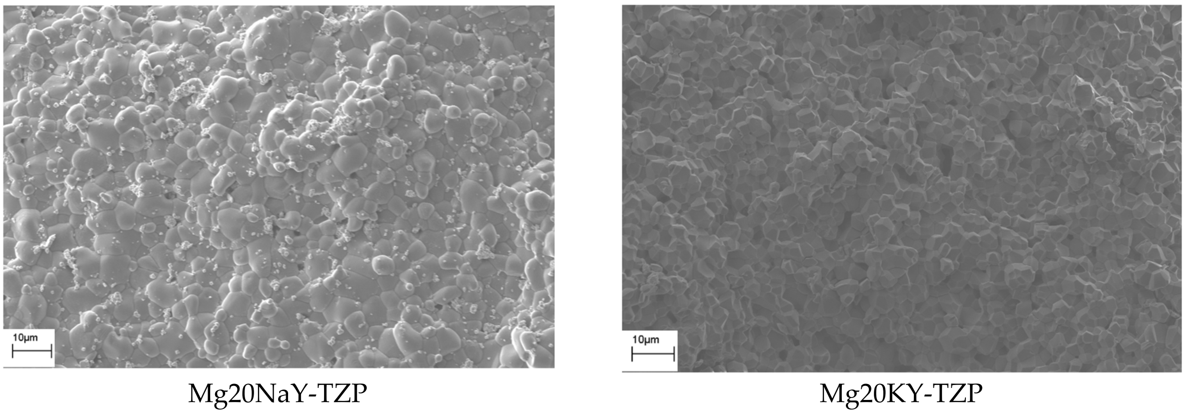

Figure S3. SEM images of the obtained ceramics.

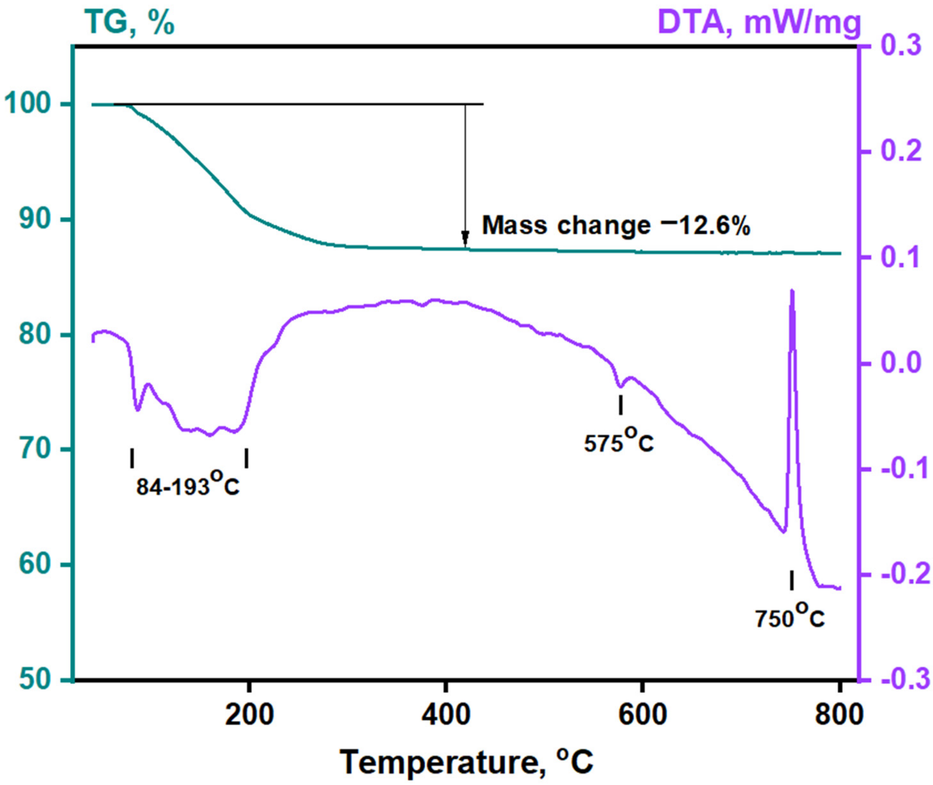

Figure S4. TG/DTA data for Mg20KY-TZP.
